# A Serum MicroRNA Panel as Potential Biomarkers for Hepatocellular Carcinoma Related with Hepatitis B Virus

**DOI:** 10.1371/journal.pone.0107986

**Published:** 2014-09-19

**Authors:** Youwen Tan, Guohong Ge, Tengli Pan, Danfeng Wen, Li Chen, Xuejun Yu, Xinbei Zhou, Jianhe Gan

**Affiliations:** 1 Department of Hepatosis, The Third Hospital of Zhenjiang Affiliated Jiangsu University, Zhenjiang, China; 2 Department of Infectious Diseases, The First Affiliated Hospital of Soochow University, Suzhou, China; The University of Hong Kong, China

## Abstract

**Background:**

The identification of new high-sensitivity and high-specificity markers for HCC are essential. We aimed to identify serum microRNAs (miRNAs) as biomarkers to be used in diagnosing hepatitis B virus (HBV) –related hepatocellular carcinoma (HCC).

**Methods:**

We investigated serum miRNA expression in (261 HCC patients, 233 cirrhosis patients, and 173 healthy controls), recruited between August 2010 and June 2013. An initial screening of miRNA expression by Illumina sequencing was performed using serum samples pooled from HCC patients and controls. Quantitative reverse-transcriptase polymerase chain reaction (qRT-PCR) was used to evaluate the expression of selected miRNAs. A logistic regression model was constructed using a training cohort (n = 357) and then validated using an independent cohort (n = 241). The area under the receiver operating characteristic curve (AUC) was used to evaluate the accuracy of the use of the biomarkers for disease diagnosis.

**Results:**

We identified 8 miRNAs (hsa-miR-206, hsa-miR-141-3p, hsa-miR-433-3p, hsa-miR-1228-5p, hsa-miR-199a-5p, hsa-miR-122-5p, hsa-miR-192-5p, and hsa-miR-26a-5p) and constructed an miRNA set that provided high diagnostic accuracy for HCC (AUC = 0.887 and 0.879 for training and validation sets, respectively). The miRNAs could also be used to differentiate HCC patients from healthy (AUC = 0.893) and cirrhosis (AUC = 0.892) patients.

**Conclusions:**

We identified a serum of miRNA panel that has considerable clinical value in HCC diagnosis.

## Introduction

Hepatocellular carcinoma (HCC) is currently the third leading cause of cancer-related deaths in the world, with mortality rates reaching up to 500,000 deaths per annum. Patients with HCC show the shortest survival time among patients with different forms of cancer, with most patients dying within 12 months of developing the tumour [1]. A previous study has suggested that early diagnosis of HCC and effective treatment are likely to prolong the lifetime of liver cancer patients [2]. Current methods for the diagnosis of HCC fall into two main categories: imaging and biomarker tests. However, the diagnostic performance of these methodsis unsatisfactory, particularly for the diagnosis of early-stage HCC. Currently, only 30% to 40% of patients with HCC are found eligible for potentially curative intervention at diagnosis, due to late clinical presentation and the lack of effective early-detection measures. Therefore, the identification of new markers with high sensitivity and specificity for HCC is the need of the hour.

MicroRNAs (miRNAs) are an emerging class of highly conserved, non-coding small RNAs that regulate gene expression at the post-transcriptional level. It is now clear that miRNAs can potentially regulate every aspect of cellular activity, including differentiation and development, metabolism and proliferation; they also play a role in regulating apoptotic cell death, cellular responses to viral infection, and tumorigenesis [3]. Recent studies provide clear evidence that miRNAs are abundant in the liver and modulate a diverse spectrum of liver functions [4]. Circulating miRNAs are extremely stable and protected from RNAase-mediated degradation in body fluids; they, therefore, have emerged as candidate biomarkers for many diseases [5], [6], [7]. The use of miRNAs as noninvasive biomarkers is of particular interest in diagnosis of liver diseases [8], [9], [10].

Many studies have demonstrated that miRNA expression profiles in HCC and non-tumor tissue are significantly different [11], [12], [13], [14], [15]. In fact, differential expression of several microRNAs in the serum, including miR-16, miR-122, miR-21, miR-223, miR-25, miR-375, and let-7f in patients with HCC, patients with hepatitis B, and healthy individuals has been reported recently [16], [17]. However, those studies had one or more of the following limitations: Limited number of screened miRNAs, small sample size, failure to differentiate HCC from hepatitis B virus (HBV) infection, and lack of independent validation.

In our study, we investigated miRNA expression profiles with independent validation in a large cohort of participants, in order to identify a set of miRNAs for the diagnosis of HCC. The cohort included healthy individuals and patients with cirrhosisand HCC related to HBV.

## Materials and Methods

### Ethics statement

The study was approved by the Medical Ethics Committee of The First Affiliated Hospital of Soochow University and The Third Hospital Affiliated to Jiangsu University (No. 2012046 and No. 272), and written informed consent was obtained from each patient prior to participation. The study was conducted in accordance with the Declaration of Helsinki.

**Table 1:** Characteristics of study subjects in the three datasets.

Variable	screening set		training set		validation set		statistics	*p*
	No.	%	No.	%	No.	%		
healthy count	n = 20		n = 90		n = 60			
age								
mean	40.25		40.2		41.43		*F = *0.569	0.567^a^
SD	7.57		6.61		7.76			
sex								
male	15	75	64	71.1	42	70	*x^2^ = *0.183	0.912^b^
female	5	25	26	28.9	18	30		
ALT								
<40 U/L	20	100	90	100	60	100	match	
AFP								
<400 ng/ml	20	100	90	100	60	100	match	
								
cirrhosis count	n = 20		n = 132		n = 78			
age								
mean	44.4		40.8		43.97		*F = *2.907	0.057^a^
SD	12.03		9.44		10.81			
sex								
male	14	70	88	66.7	46	59	*x^2^ = *1.57	0.456^b^
female	6	30	44	33.3	32	41		
ALT								
<40 U/L	14	70	66	50	47	60.3	*x^2^ = *4.021	0.134^b^
≥40 U/L	6	30	66	50	31	39.7		
AFP								
<400 ng/ml	20	100	131	99.2	77	99.1	*x^2^ = *0.913	1^c^
≥400 ng/mL	0	0	1	0.8	1	0.9		
								
HCC count	n = 20		n = 135		n = 103			
age								
mean	49.8		53.57		52.01		*F = *1.311	0.271^a^
SD	10.85		11.59		10.21			
sex								
male	16	80	112	83	76	73.8	*x^2^ = *2.984	0.225^b^
female	4	20	23	17	27	26.2		
ALT								
<40 U/L	11	55	47	34.8	44	42.7	*x^2^ = *3.696	0.158^b^
≥40 U/L	9	45	88	65.2	59	57.3		
AFP								
<400 ng/ml	15	75	84	62.2	62	60.2	*x^2^ = *1.569	0.456^b^
≥400 ng/mL	5	25	51	37.8	41	39.8		

a One-Way ANOVA,

b Pearson Chi-Square,

c Fisher’s Exact Test.

### Study design, patients, and healthy controls

**Figure 1:**
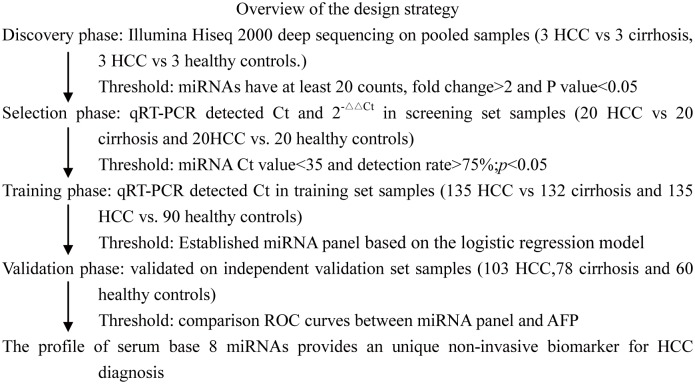
A flow-chart of the experimental design.

A multistage, case-control study was designed to identify a serum miRNA profile as a surrogate marker for HCC (Fig. 1). A total of 261 HCC patients, 233 cirrhosi patients and 173 healthy controls were enrolled in our study. In the discovery biomarker stage, 9 serum samples pooled from 3 healthy control donors, 3 HCC patients and 3 cirrhosi patients treated at The First Affiliated Hospital of Soochow University were subjected to Illumina Hiseq 2000 deep sequencing to identify the miRNAs that were significantly differentially expressed. In the biomarker selection stagedifferent expression miRNAs were validated by qRT-PCR in 20 HCC patients, 20 cirrhosis patients and 20 healthy controls. Subsequently, 135 HCC patients, 132 cirrhosis patients and 90 healthy controls (from The First Affiliated Hospital of Soochow University and The Third Hospital of Zhenjiang Affiliated Jiangsu University) formed a training set. Sequential validation was performed using a hydrolysis probe-based qRT-PCR assay to refine the number of serum miRNAs as an HCC signature. Whereas an additional 103 HCC patients, 78 cirrhosis patients and 60 healthy controls serum samples (from The Third Hospital of Zhenjiang Affiliated Jiangsu University) formed an independent validation set. All patients were diagnosed with HCC and cirrhosis between August 2010 and June 2013, and blood samples were collected prior to any therapeutic procedure.

Chronic HBV infection was defined as positivity for HBV surface antigen for at least 6 months, positivity for HBV DNA by PCR analysis, and HBV infection-compatible results in a liver biopsy. All patients were positive for HBsAg and did not have any other types of liver diseases such as chronic hepatitis C, alcoholic liver diseases, autoimmune liver diseases, or metabolic liver diseases. The diagnosis of HCC and cirrhosis was histopathologically confirmed. Data on all subjects were obtained from medical records, pathology reports and personal interviews with the subjects. Tumor-free healthy control subjects were recruited from a large pool of individuals seeking a routine health check-up at the Healthy Physical Examination Centre of The First Affiliated Hospital of Soochow University who showed no evidence of disease. The demographics and clinical features of the patients arelisted in Table. 1.

**Table 2:** Differentially expressed miRNAs in HCC and healthy groups.

no.	miR_name	fold change	fold change (log2)	up/down	miR_seq
1	hsa-miR-190b_R+1	12.4564	3.6388	up	UGAUAUGUUUGAUAUUGGGUUU
2	hsa-miR-141-3p	6.8951	2.7856	up	UAACACUGUCUGGUAAAGAUGG
3	hsa-miR-4532_R+2	6.6370	2.7305	up	CCCCGGGGAGCCCGGCGCG
4	hsa-mir-6127-p3	4.6459	2.2160	up	UGAGGGAGUGGGUGGGAGG
5	hsa-miR-99b-3p_R−2	4.5838	2.1965	up	CACCCGUAGAACCGACCUUG
6	hsa-miR-1228-5p	4.4131	2.1418	up	GUGGGCGGGGGCAGGUGUGUG
7	hsa-miR-30a-3p	0.4634	−1.1098	down	UGUAAACAUCCUCGACUGGAAG
8	hsa-miR-199a-5p	0.4452	−1.1673	down	ACAGUAGUCUGCACAUUGGUUA
9	hsa-let-7f-5p	0.3459	−1.5315	down	UGAGGUAGUAGAUUGUAUAGUU
10	hsa-miR-122-5p	0.3405	−1.5541	down	UGGAGUGUGACAAUGGUGUUUG
11	hsa-miR-192-5p	0.2755	−1.8601	down	CUGACCUAUGAAUUGACAGCC
12	hsa-miR-98-5p	0.2603	−1.9419	down	UGAGGUAGUAAGUUGUAUUGUU
13	hsa-miR-574-3p	0.1954	−2.3556	down	UGAGUGUGUGUGUGUGAGUGUGU
14	hsa-miR-30e-3p	0.1895	−2.3999	down	CUUUCAGUCGGAUGUUUACAGC
15	hsa-miR-6852-5p	0.0739	−3.7583	down	CCCUGGGGUUCUGAGGACAUG

**Table 3:** Differentially expressed miRNAs in HCC and cirrhosis groups.

no.	miR_name	fold change	fold change(log2)	up/down	miR_seq
1	hsa-miR-206	12.1198	3.5993	up	UGGAAUGUAAGGAAGUGUGUGG
2	hsa-mir-1285-1-p5	7.2943	2.8668	up	GAUCUCACUUUGUUGCCCAGG
3	hsa-miR-10a-5p	6.2059	2.6336	up	UACCCUGUAGAUCCGAAUUUGUG
4	hsa-miR-511-5p	5.8074	2.5379	up	GUGUCUUUUGCUCUGCAGUCA
5	hsa-miR-433-3p	5.6166	2.4897	up	AUCAUGAUGGGCUCCUCGGUGU
6	hsa-miR-100-5p_R−1	0.4026	−1.3125	down	AACCCGUAGAUCCGAACUUGUG
7	hsa-miR-483-5p_R−1	0.4002	−1.3213	down	AAGACGGGAGGAAAGAAGGGAG
8	hsa-miR-584-5p_R−1	0.3461	−1.5308	down	UUAUGGUUUGCCUGGGACUGG
9	hsa-miR-28-5p_R−2	0.2386	−2.0672	down	AAGGAGCUCACAGUCUUGAG
10	hsa-miR-30b-5p	0.2379	−2.0716	down	UGUAAACAUCCUACACUCAGCU
11	hsa-miR-30c-5p	0.1856	−2.4300	down	UGUAAACAUCCUACACUCUCAGC
12	hsa-miR-26a-5p	0.1677	−2.5757	down	UUCAAGUAAUCCAGGAUAGGCU
13	hsa-miR-4454_L-2	0.1593	−2.6498	down	GGAUCCGAGUCACGGCACCA
14	hsa-let-7e-5p	0.1351	−2.8884	down	UGAGGUAGGAGGUUGUAUAGUU
15	hsa-let-7c-5p	0.1275	−2.9709	down	UGAGGUAGUAGGUUGUAUGGUU
16	hsa-miR-4433b-5p	0.0891	−3.4882	down	AUGUCCCACCCCCACUCCUGU
17	hsa-miR-455-5p	0.0681	−3.8764	down	UAUGUGCCUUUGGACUACAUCG

### RNA isolation and library preparation

About 5 mL of venous blood was collected from each participant. The whole blood was separated into serum and cellular fractions by centrifugation at 4,000 rpm for 10 min, followed by 5 min centrifugation at 13,000 rpm for complete removal of cell debris. The supernatant serum was stored at −80°C until analysis. Total RNA was isolated using LCS TRK1001 miRNeasy kit (LC Sciences, Hangzhou, China). The libraries were constructed from total RNA using the Illumina Truseq Small RNA Sample Preparation Kit (Illumina, San Diego, CA, USA) according to the manufacturer’s protocol. Briefly, RNA 3′ (P-UCGUAUGCCGUCUUCUGCUUG-UidT) and 5′ (GUUCAGAGUU CUACAGUCCGACGAUC) adapters were ligated to target miRNAs in two separate steps. Reverse transcription reaction was applied to the ligation products to create single stranded cDNA. The cDNA was amplified by PCR using a common primer and a primer containing the index sequence (CAAGCAGAAGACGGCATACGA). The quantity and purity of total RNAs were monitored using a NanoDrop ND-1000 spectrophotometer (NanoDrop Inc, Wilmington, DE, USA) at a 260/280 ratio >2.0. The integrity of total RNAs was analyzed using an Agilent 2100 Bioanalyzer system and RNA 6000 Nano LabChip Kit (Agilent Tech, Santa Clara, CA, USA) with RNA integrity number >8.0. Finally, Illumina sequencing technology was employed to sequence these prepared samples.

**Table 4:** Expression profiles of 32 candidate miRNA on qRT-PCR in screening set.

no.	miR_name	HCC versuscontrol	HCC versushealthy		HCC versuscirrhosis	
		p value	p value	fold change	p value	fold change
1	hsa-miR-190b_R+1	0.2900	0.3500	0.9800	0.108	3.6709
2	**hsa-miR-206**	0.0030	0.0060	9.9350	0.03	3.5094
3	hsa-mir-1285-1-p5	0.0530	1.0000	1.1356	0.108	1.7391
4	**hsa-miR-141-3p**	0.0000	0.0000	11.0410	0.0000	5.8240
5	hsa-miR-4532_R+2	ND	ND	ND	ND	ND
6	hsa-miR-10a-5p	0.2600	0.2890	1.9650	0.602	1.3910
7	hsa-miR-511-5p	0.1010	0.2890	3.6600	0.108	2.8090
8	**hsa-miR-433-3p**	0.0000	0.0060	3.1180	0.004	4.1835
9	hsa-mir-6127-p3	ND	ND	ND	ND	ND
10	**hsa-miR-1228-5p**	0.0140	0.6020	3.1970	0.782	1.8180
11	hsa-miR-99b-3p_R−2	0.1000	1.0000	1.7900	0.031	2.0200
12	hsa-miR-30a-3p	ND	ND	ND	ND	ND
13	**hsa-miR-199a-5p**	0.0000	0.0000	0.5818	0.0001	0.8745
14	hsa-miR-100-5p_R−1	0.0950	0.0300	0.9230	0.108	1.0251
15	hsa-miR-483-5p_R−1	ND	ND	ND	ND	ND
16	hsa-miR-584-5p_R−1	0.0970	0.0900	0.7550	0.723	0.7840
17	hsa-let-7f-5p	0.1430	0.1080	0.8180	0.602	0.9250
18	**hsa-miR-122-5p**	0.0000	0.0000	0.2735	0.0000	0.5409
19	**hsa-miR-192-5p**	0.0010	0.0060	0.7650	0.012	0.8460
20	hsa-miR-98-5p	0.2643	0.2890	0.7714	0.122	0.8330
21	hsa-miR-28-5p_R−2	0.2386	0.1080	0.9360	0.289	0.9260
22	hsa-miR-30b-5p	0.0970	0.0300	0.9020	0.125	0.8740
23	hsa-miR-574-3p	0.1954	0.6020	1.2540	0.268	0.9190
24	hsa-miR-30e-3p	ND	ND	ND	ND	ND
25	hsa-miR-30c-5p	ND	ND	ND	ND	ND
26	**hsa-miR-26a-5p**	0.0010	0.0060	0.6530	0.0008	0.5370
27	hsa-miR-4454_L-2	ND	ND	ND	ND	ND
28	hsa-let-7e-5p	0.0980	0.1830	1.0130	0.0734	0.8360
29	hsa-let-7c-5p	0.2690	0.2470	0.8530	0.0772	0.7630
30	hsa-miR-4433b-5p	0.0891	0.1320	0.7640	0.112	0.7780
31	hsa-miR-6852-5p	ND	ND	ND	ND	ND
32	hsa-miR-455-5p	0.2250	0.3520	0.9740	0.074	0.6840

ND:not determined, miRNA Ct value >35 and detection rate <75%.

### Illumina sequencing and data analysis

The raw sequences were processed using the Illumina pipeline program. After masking of adaptor sequences and removal of contaminated reads, the clean reads were filtered for miRNA prediction with the software package ACGT101-miR-v4.2 (LC Sciences, Houston, Texas, USA) and subsequently analyzed according to report [18]. Secondary structure prediction of individual miRNAs was performed by Mfold software (Version 2.38; http://mfold.rna.albany.edu/?q=mfold/RNA-Folding-Form) using the default folding conditions. The raw dates were reduced to cleaned sequences by removal of the following sequences: (1) 3ADT&length filter: reads were removed due to 3ADT not being found, and reads with length <18 and >26 were removed. (2) Junk reads: Junk: ≥2N, ≥7A, ≥8C, ≥6G, ≥7T, ≥10 Dimer, ≥6 Trimer, or ≥5 Tetramer. (3) Rfam: Collection of many common non-coding RNA families except miRNAs (http://rfam.janelia.org). (4) Repeats: Prototypic sequences representing repetitive DNA from different eukaryotic species (http://www.girinst.org/repbase). (5) Notes: There was overlap in mapping of reads with mRNA, rRNA, tRNA, snRNA, snoRNA, and repeats. (6) mRNA Database: (http://www.ncbi.nlm.nih.gov/). The clean sequence reads were mapped with miRBase 20.0, allowing a mismatch of one or two nucleotide bases. More detailed description of the computational pipeline employed for data handling is reported in a flow-chart outline of study procedures (Figure. S1). All data were transformed to log base 2. Differences between the samples were calculated using chi-square and fisher’s exact test. Only miRNAs with fold difference >2.0 and *P*<0.05 were considered statistically significant.

**Table 5:** Expression profiles of 8 candidate miRNA from qRT-PCR in training set.

no.	miR_name	HCC versuscontrol	HCC versushealthy		HCC versuscirrhosis	
		p value	p value	fold change	p value	fold change
1	**hsa-miR-206**	0.0010	0.0000	9.8846	0.0000	4.7465
2	**hsa-miR-141-3p**	0.0000	0.0000	8.4126	0.0000	7.5844
3	**hsa-miR-433-3p**	0.0000	0.0060	7.0355	0.737	3.2273
4	**hsa-miR-1228-5p**	0.0245	0.1452	2.8560	0.315	3.4431
5	**hsa-miR-199a-5p**	0.0000	0.2450	0.9462	0.0000	0.9104
6	**hsa-miR-122-5p**	0.0000	0.0001	0.6942	0.0000	0.8349
7	**hsa-miR-192-5p**	0.0001	0.0006	0.9218	0.0000	0.8958
8	**hsa-miR-26a-5p**	0.0000	0.0001	0.7231	0.0000	0.4831

ND:not determined, miRNA Ct value >35 and detection rate <75%.

### qRT-PCR validation study and data analysis

qRT-PCR-based relative quantification of miRNAs (300 µL of serum from each participant) was performed with SYBR Premix Ex Taq (TaKaLa) according to the manufacturer’s instructions using a Rotor-Gene 3000 Real-time PCR machine (Corbett Life Science, Sydney, Australia). According to the results obtained, miRNA-24 has been reported to be consistently present in human serum [19], [20]. Moreover, our previous experience is that miRNA-24 maintains a stable expression, and that the level of miRNA-24 served as an internal control in serum miRNA relative quantitative analysis. The specificity of each PCR product was validated by melting curve analysis at the end of PCR cycles. All samples were analyzed in triplicate, and the cycle threshold (Ct) value was defined as the number of cycles required for the fluorescent signal to reach the threshold. The relative expression levels of miRNAs in serum were calculated using the formula 2^−ΔΔCt^ where ΔΔCt = [Ct (target, test) − Ct (ref, test)] − [Ct (target, calibrator) − Ct (ref, calibrator)]. All primers used were obtained from Invitrogen company (Shanghai, China).

### Statistical analysis

All Illumina sequencing data were transformed to log base2. Differences between the samples were calculated using chi-square and fisher’s exact test. Only miRNAs with fold difference >2.0 and *P*<0.05 were considered statistically significant. Data were presented as median ± SD. The data of demographic and clinical features of the HCC patients and healthy controls were analyzed using the statistical Package for the Social Sciences(SPSS) version 21.0 software (SPSS Inc, Chicago, IL, USA). For the data 2^−ΔΔCt^ of miRNAs obtained by qRT-PCR, Mann-Whitney unpaired test was used to compare between HCC patients and controls. A stepwise logistic regression model was used to select diagnostic miRNA markers based on the training dataset. The predicted probability of being diagnosed with HCC was used as a surrogate marker to construct the receiver operating characteristic (ROC) curve. Area under the ROC curve (AUC) was used as an accuracy index for evaluating the diagnostic performance of the selected miRNA panel. The ROC and regression analysis was performed using the software 21 MedCalc (Version 10.4.7.0; MedCalc, Mariakerke, Belgium). All *P*-values were two-sided.

## Results

### Description and clinical features of patients

The characteristics of the study participants are presented in Table. 1. There was no significant difference in the distribution of age, sex, and alanine aminotransferase (ALT) expression among the three groups (healthy, cirrhosis, and HCC).

### Global analysis of miRNAs by deep sequencing

**Figure 2:**
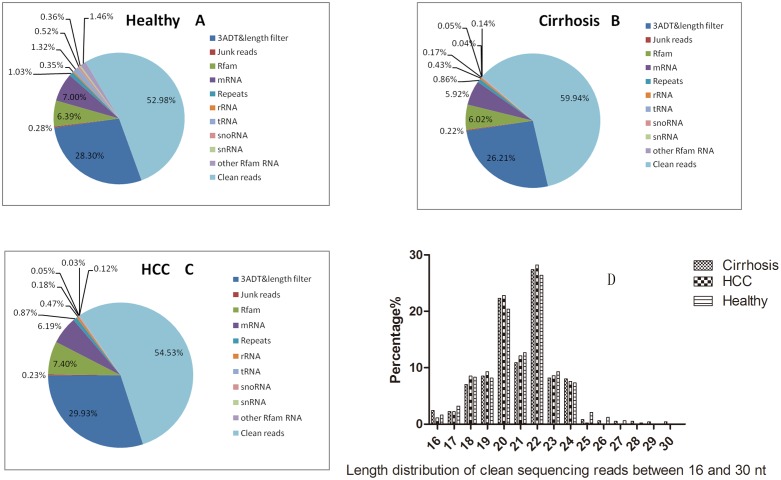
Sequenced reads and the distribution of reads. The Illumina Hiseq 2000 sequencing of the microRNAs obtained from the sera of patients in the control group, carrier group, and CHB group, produced 9,364,754,10,491,694, and 7,896,608 raw-reads, respectively, which, after extensive preprocessing and quality control, were reduced to 459,890,859,216, and 494,523 clean reads (Fig. 2A–C). All the distribution of reads of 16–30 nt length is presented in Fig. 2D.

Illumina HiSeq 2000 sequencing of the miRNAs obtained from the sera of patients in the healthy control group, cirrhosis group and HCC group produced 9,364,754, 10,491,694, and 7,896,608 raw-reads, respectively, which, after extensive preprocessing and quality control, were reduced to 459,890, 859,216, and 494,523 clean reads (Fig. 2A–C, Table. S1). Distribution of reads of 16–30 nt length is presented in (Fig. 2D). In our study, we found that the length of miRNA is generally 20 to 22 nt. Clean reads were mapped to human miRNA (miRs) database v20.0 (ftp://mirbase.org/pub/mirbase/CURRENT/), pre-miRNA (mirs) database v20.0 (ftp://mirbase.org/pub/mirbase/CURRENT/), and genome database (ftp.ncbi.nih.gov/genomes/H sapiens/Assembled chromosomes/seq/). A total of 2,754 unique reads map to human miRNAs or pre-miRNAs in miRbase and the pre-miRNAs further map to the human genome and expressed sequence tags.

### Analysis of differentially expressed miRNAs

We normalized the differential expression of miRNA count data, and the number of individual miRNA reads was standardized by the total numbers of 1,000,000 reads in each sample. Comparing the HCC and healthy control groups, the differential expression levels of 143 miRNAs have significant differences. Among them, 6 miRNAs were up-regulated (fold change >4-fold, *P*<0.05) in the control group, 9 down-regulated (fold change >2-fold, *P*<0.05), shown in Table. 2. Comparing the HCC and cirrhosis groups, differential expression levels of 84 miRNAs have significant differences. Among them, 5 miRNAs were up-regulated (fold change >4-fold, *P*<0.05) in cirrhosis group, 12 down-regulated (fold change >2-fold, *P*<0.05), shown in Table. 3.

### Differential Expression Profile of Eight Selected miRNAs

We used qRT-PCR assay to confirm the expression of 32 candidate miRNAs that were selected from the previous step from an independent cohort of 60 serum samples. Threshold levels were found to be as follows: MiRNA Ct<35 and detection rate >75%. We determined the 2^−ΔΔCt^ of 32 candidate miRNAs in three groups, Mann-Whitney unpaired test was used to compare between HCC patients and controls. Eight of the 32 miRNAs had significantly different expression levels between the HCC and control groups (healthy + cirrhosis group), as shown in Table. 4. These were hsa-miR-206, hsa-miR-141-3p, hsa-miR-433-3p, hsa-miR-1228-5p, hsa-miR-199a-5p, hsa-miR-122-5p, hsa-miR-192-5p, and hsa-miR-26a-5p.

### MiRNA expression profile for HCC patients versus control patients in the training cohort

**Figure 3:**
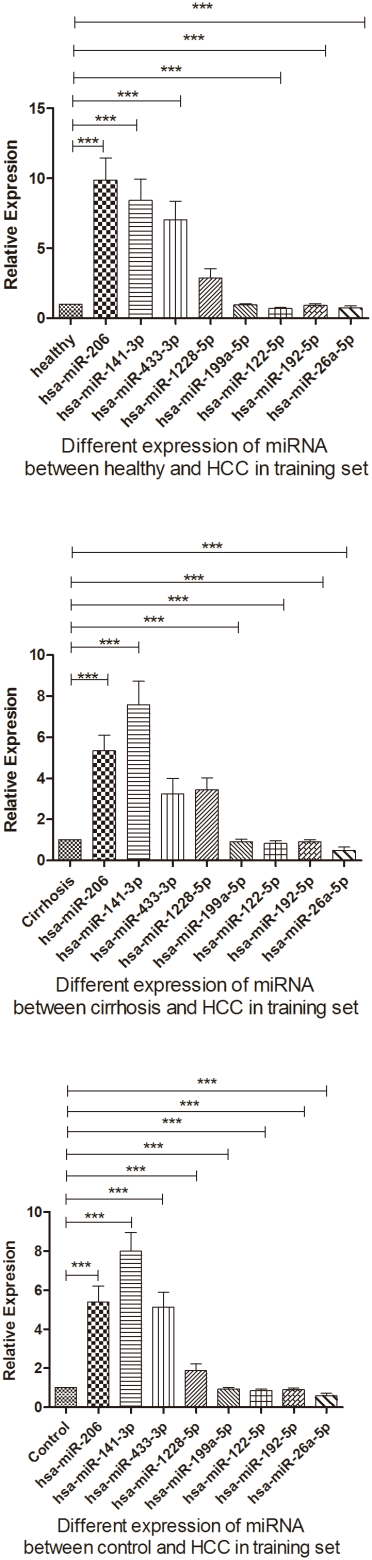
Differential expression of microRNAs in the training set.

**Figure 4:**
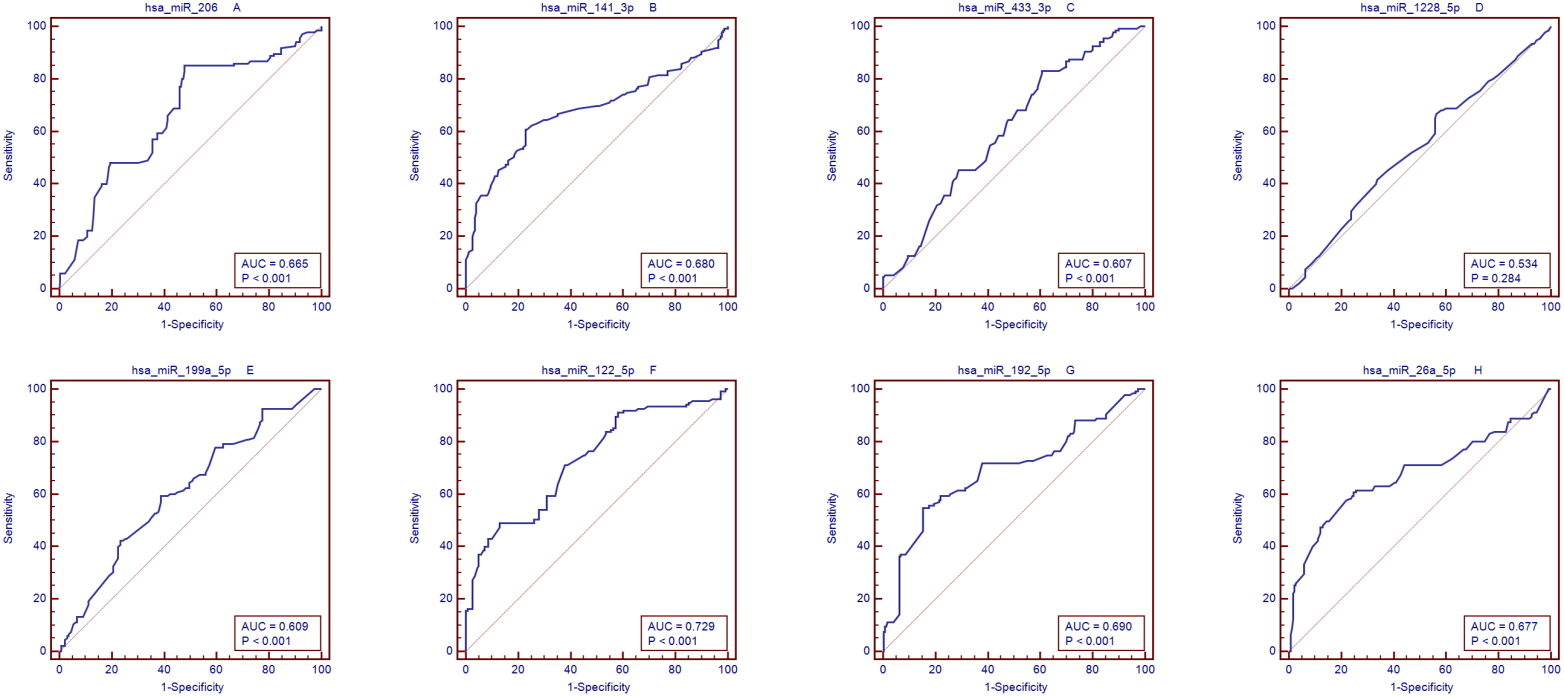
AUC of microRNAs for control subjects and HCC patients. Area under the curve (AUC) for the microRNAs (miRs). A:hsa-miR-206,B:hsa-miR-141-3p,C:hsa-miR-433-3p,D:hsa-miR-1228-5p,E:hsa-miR-199a-5p,F:hsa-miR-122-5p,G:hsa-miR-192-5p, and H:hsa-miR-26a-5p.

We used qRT-PCR assay to confirm the expression of 8 candidate miRNAs that were selected from the previous step. There were 6 miRNAs with significantly different expression between HCC and healthy groups, as shown in (Fig. 3A and Table. 5), and 6 other miRNAs showed significantly different expression between HCC and cirrhosis groups (Fig. 3B and Table. 5). We identified 8 of these miRNAs that showed significantly different expression when compared with the control group (Fig. 3C and Table. 5); These were then selected for the next validation. These were hsa-miR-206, hsa-miR-141-3p, hsa-miR-433-3p, hsa-miR-1228-5p, hsa-miR-199a-5p, hsa-miR-122-5p, hsa-miR-192-5p, and hsa-miR-26a-5p. Compared to Ct of their levels in the control samples, the diagnostic accuracy using these miRNAs, as measured by AUC, was 0.665,0.68, 0.607, 0.534,0.609,0.729,0.69 and 0.677, respectively (Table. S2–4, Fig. 4A–H).

### Establishing the predictive miRNA panel for HCC versus control

**Figure 5:**
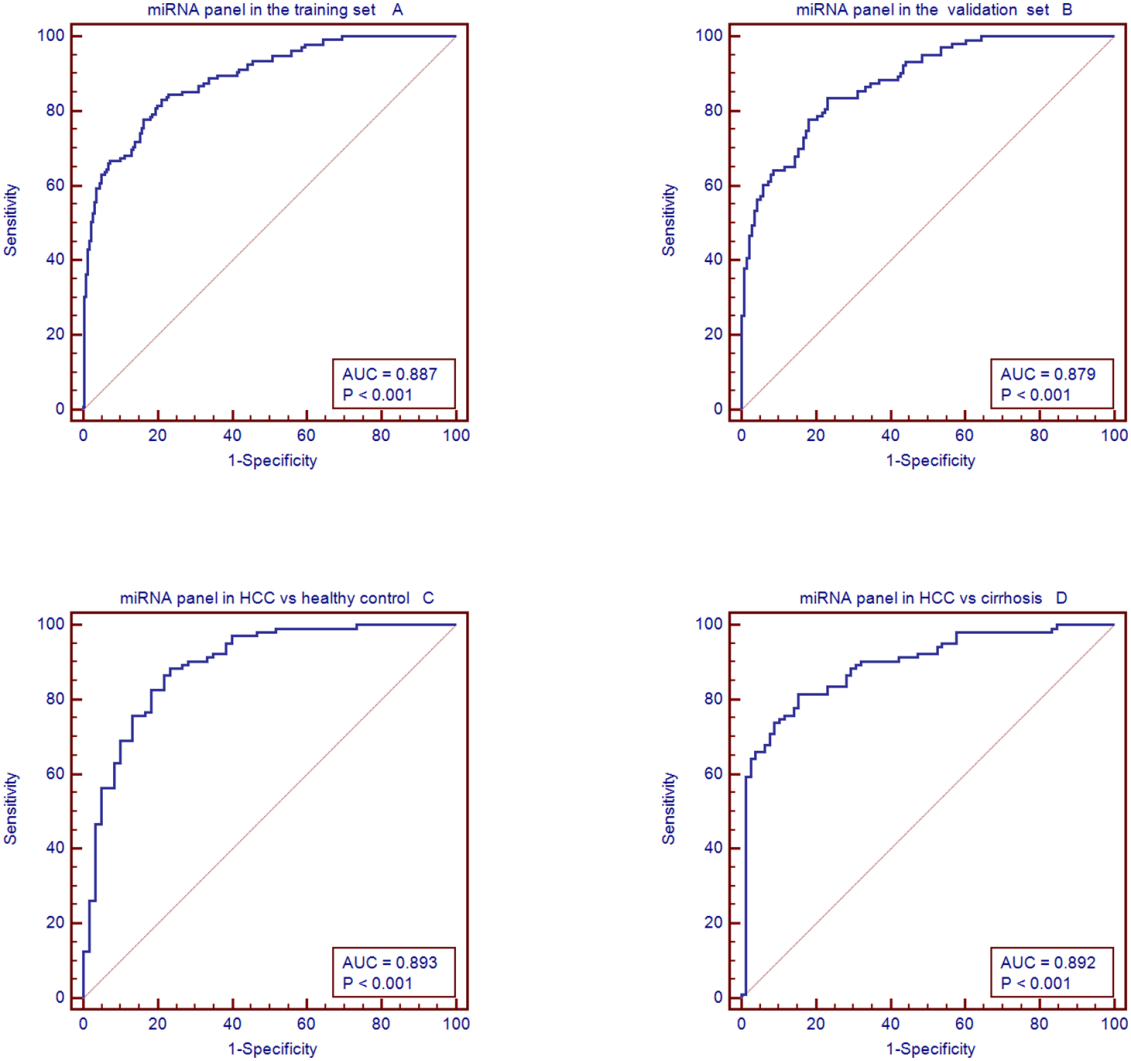
The diagnosis value of miRNA panel. A:The AUC for the microRNAs panel in the training set; B: the AUC of the microRNAs panel in the validation set; C: is AUC of the microRNAs panel used to distinguish HCC patients from healthy patients, and D is AUC of the microRNAs panel used to distinguish HCC patients from cirrhosis patients.

A stepwise logistic regression model was applied on the training data set to estimate the chances of being diagnosed with HCC. All of the 8 miRNAs turned out to be significant predictors. The predicted probability of being diagnosed with HCC from the logit model based on the 8-miRNA panel (Table S5), logitP = −11.8472 + 0.52147miR122 − 0.22949miR1228 − 0.27621miR141 + 0.34063miR192 + 0.33325mi199a − 0.30556miR206 + 0.40777miR26a − 0.38006miR433, was used to construct the ROC curve. The diagnostic performance for the established miRNA panel was evaluated using ROC analysis. The AUC for the miRNA panel was 0.887 (95% CI = 0.850 to 0.918), sensitivity = 85.55%, specificity = 73.3%, Fig. 5A).

### Validating the miRNA panel

The parameters estimated from the training data set were used to predict the probability of being diagnosed with HCC for the independent validation data set (251 serum samples). Similarly, the predicted probability was used to construct the ROC curve. The AUC of the miRNA panel was 0. 879 (95% CI = 0.842–0.941; sensitivity = 90.3%, specificity = 76.2%, Fig. 5B).

The performance of the miRNA panel in differentiating the HCC group from the healthy as well as the cirrhosis groups was also evaluated. The analysis demonstrated that the miRNAs had a high accuracy in distinguishing HCC patients from healthy patients (AUC = 0.893; 95% CI, 0.849 to 0.94; sensitivity 82.8%; specificity 83.3%, Fig. 5C) and cirrhosis patients (AUC = 0.892; 95% CI, 0.844 to 0.939; sensitivity 81.6%; specificity 84.6%. Fig. 5D).

### Comparison of the AUC of the miRNA panel with that of AFP in the validation set

Using the same serum samples, we evaluated the AUC of the AFP in different groups. The analysis demonstrated that the AFP also had a high accuracy in distinguishing HCC from healthy patients (AUC = 0.844; 95% CI, 0.785 to 0.902; sensitivity 60.2%; specificity 100%), cirrhosis patients (AUC = 0.708; 95% CI, 0.632 to 0.783; sensitivity 57.3%, specificity 79.5%) and control subjects (AUC = 0.766; 95% CI, 0.703 to 0.829; sensitivity 59.2%, specificity 87%).

**Figure 6:**
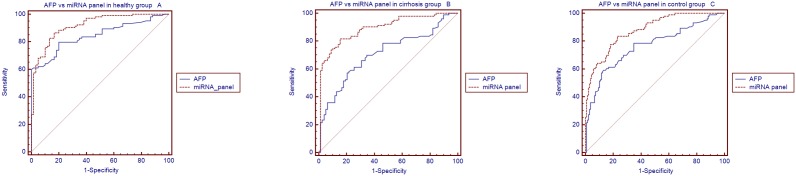
Comparison of AUC of the microRNA panel with that of AFP in the validation set. A:In healthy group;B:In cirrhosis group, and C:In control group.

We also compared the AUC of the miRNA panel with that of AFP. There was no difference between the AUC values of the miRNA panel and those of AFP (difference between areas = 0.0735, 95% CI = 0.000145 to 0.148, *P* = 0.514, Fig. 6A) in the healthy group. However, there were significant differences between the AUC values of the miRNA panel and those of AFP in the cirrhosis group (difference between areas = 0.184, 95% CI = 0.0925 to 0.276, *P* = 0.0001, Fig. 6B) and control group (difference between areas = 0.113, 95% CI = 0.0344 to 0.192, *P* = 0.0049, Fig. 6C).

## Discussion

Sensitive and specific cancer biomarkers are essential for early detection and diagnosis of HCC, as well as for developing preventive screening. However, current methods are insufficient to detect HCC in the early stages. Advances in magnetic resonance imaging and computed tomography have greatly improved imaging of focal hypervascular masses consistent with HCC, but these procedures are costly and not readily available in developing countries. Laboratory data including serum alfa-fetoprotein (AFP) and des-gamma carboxyprothrombin (DCP) levels have been used as HCC biomarkers for a long time. However, the accuracy of AFP is modest (sensitivity: 39–65%; specificity: 76–94%). One-third of cases of early-stage HCC (tumors <3 cm) are missed using AFP analysis [21], and serum AFP levels are also elevated in patients with benign liver diseases, such as hepatitis and cirrhosis [22], [23].

Many miRNAs are dysregulated in HCC; thus, it is to be expected that circulating miRNA levels are also affected by HCC progression. The high stability of miRNAs in circulation makes them perfect biomarkers, especially for detection of early stage, presymptomatic disease [24]. It is interesting that circulating miR-21 [16], [25], miR-222 [25], and miR-223 [26] were found to be upregulated in the serum/plasma of HCC patients associated with HBV or HCV.

Downregulation of subsets of miRNAs is a common finding in HCC, suggesting that some of these miRNAs may act as putative tumor suppressor genes. Restoration of tumor suppressive miRNAs leads to cell cycle block, increased apoptosis, and reduced tumor angiogenesis and metastasis by inhibiting migration and invasion. Of these miRNAs, miR-122 and miR-199 appear to be particularly important in HCC [27], [28], [29]. Liver-specific miR-122 is the most abundant miRNA in the liver and it plays an important role in regulating hepatocyte development and differentiation [30], [31]. The expression of miR-122 is downregulated in HCC tumor tissues and cancer cell lines, while its overexpression has been found to induce apoptosis and suppress proliferation in HepG2 and Hep3B cells [32]. The role of miR-122 in liver cancer has been demonstrated directly by the generation of miR-122 knockout mice [33], [34].

Our study revealed that serum hsa-miR-206, hsa-miR-141-3p, hsa-miR-433-3p, hsa-miR-1228-5p, hsa-miR-199a-5p, hsa-miR-122-5p, hsa-miR-192-5p, and hsa-miR-26a-5p were potential circulating markers for HCC diagnosis. The miRNA panel with eight miRNAs from the multivariate logistic regression model demonstrated high accuracy in HCC diagnosis. The association at the tissue level between HCC and four ofthe eight miRNAs (miR-122, miR-199, miR-192, and miR-26a) in our study has been reported previously [17], [35], [36].

At the circulating blood level, the diagnostic performance of miR-21, miR-122, and miR-223 in discriminating patients with HCC from the healthy group was reported by Xu et al [16]. However, their study failed to distinguish HCC from chronic hepatitis. Qu et al [37] found miR-16 to have moderate diagnostic accuracy of HCC, with sensitivity of 72.1% and specificity of 88.8%. In our study, miR-16 did show significant down-regulation in HCC as compared to control, but it did not meet our candidate microRNA selection criteria at the microarray level. Li et al [13] reported an extraordinarily high diagnostic accuracy of serum microRNA profiles for the diagnosis of HCC (AUC = 0.97–1.00) with miRNAs10a, 125b, 223, 23a, 23b, 342-3p, 375, 423, 92a, and 99a. However, the need for different markers for different group comparisons with different critical values in their study (HCC versus healthy, HCC versus HBV, healthy versus HBV, healthy versus HCV, and HBV versus HCV) raised concerns about the robustness of these markers. Furthermore, these results were not validated either internally or externally.

AFP is the most widely used tumor biomarker currently available for the early detection of HCC. Findings of a previous clinical study demonstrated that serum AFP had a sensitivity of 41–65% and specificity of 80–100% [38]. We found that AFP showed high accuracy in discriminating HCC patients from healthy subjects. At present, AFP measurement and ultrasound at 6-month intervals are the standard tools to screen for HCC in China. AFP is considered to be a useful and feasible tool for screening and early diagnosis in China due to its convenience, especially due to the fact that more than 60% of patients with HCC have an AFP level of >400 ng/ml [39]. However, the widely used marker AFP does not yield satisfactory results for early diagnosis of HCC, particularly AFP-negative HCC. AFP results are positive during pregnancy, as well as for active liver disease, embryonic tumor and certain gastrointestinal tumors; Furthermore, false-negative results and limitations in terms of sensitivity in different detection methods add to the limitations of this biomarker. For example, a small hepatic tumor results in AFP expression being lower than the limit of detection, whereas AFP expression is delayed or higher than the limit of detection when the tumor is large, yielding AFP-negative HCC. We compared the AUC of the miRNA panel with that of AFP. There were significant differences between the AUC values of the miRNA panel and those of AFP in the cirrhosis group. Compared with other studies of circulating miRNA in HCC diagnosis [13], [17], [23], [26], our study is unique for the following reasons: first, we screened a large number of serum miRNAs via the Illumina Hiseq 2000 sequencing method, which gave us a better chance to identify potential diagnostic markers. Secondly, we included not only HCC and healthy groups, but a cirrhosis group as well. It is well-known that the pathogenesis of HCC is heterogenous and that multiple mechanisms of tumorigenesis could be involved (tumor suppressor gene, oncogene, viral effects, angiogenesis, etc). Nonetheless, we hypothesized that, similar to the adenoma-carcinomasequence in colorectal cancer, the clinical pathway of most HBV-related HCC may follow the four stages of healthy, hepatitis, cirrhosis, and HCC. Because of the long incubation time, miRNA disturbance might occur during any of these stages (hepatitis, cirrhosis, or HCC) before the clinical/pathophysiological manifestationof HCC. Thus, all the representative differential miRNAs, namely HCC versus healthy, HCC versus hepatitis, and HCC versus cirrhosis should be considered. Failure to do so might be the source of the unsatisfactory differentiation of HCC from hepatitis or cirrhosis in other studies. Finally, the microRNA panel identified in our study was validated by a large, independent cohort from two independent medical centers.

In summary, we identified a serum microRNA panel that differentiates HCC from healthy and cirrhosis with a high degree of accuracy and validated it in a large number of subjects. Our study demonstrates that this serum microRNA panel has considerable clinical value for early diagnosis of HCC, so that more patients, who would have otherwise missed the curative treatment window, can benefit from therapy.

## Supporting Information

Figure S1 A flow-chart of study procedures.
(TIF)

Table S1 Overview of reads from raw data to cleaned sequences.
(DOCX)

Table S2 AUC of ROC curves between HCC and healthy controls in the training set.
(DOCX)

Table S3 AUC of ROC curves between HCC and cirrhosis in the training set.
(DOCX)

Table S4 AUC of ROC curves between HCC and control in the training set.
(DOCX)

Table S5 Logistic regression of miRNAs between HCC patients and control subjects in the training set.
(DOCX)
