# Supplementary figures and images for: A Serum MicroRNA Panel as Potential Biomarkers for Hepatocellular Carcinoma Related with Hepatitis B Virus
Source: PLoS One. 2014 Sep 19;9(9):e107986. doi: 10.1371/journal.pone.0107986 (PMC4169601; doi:10.1371/journal.pone.0107986)

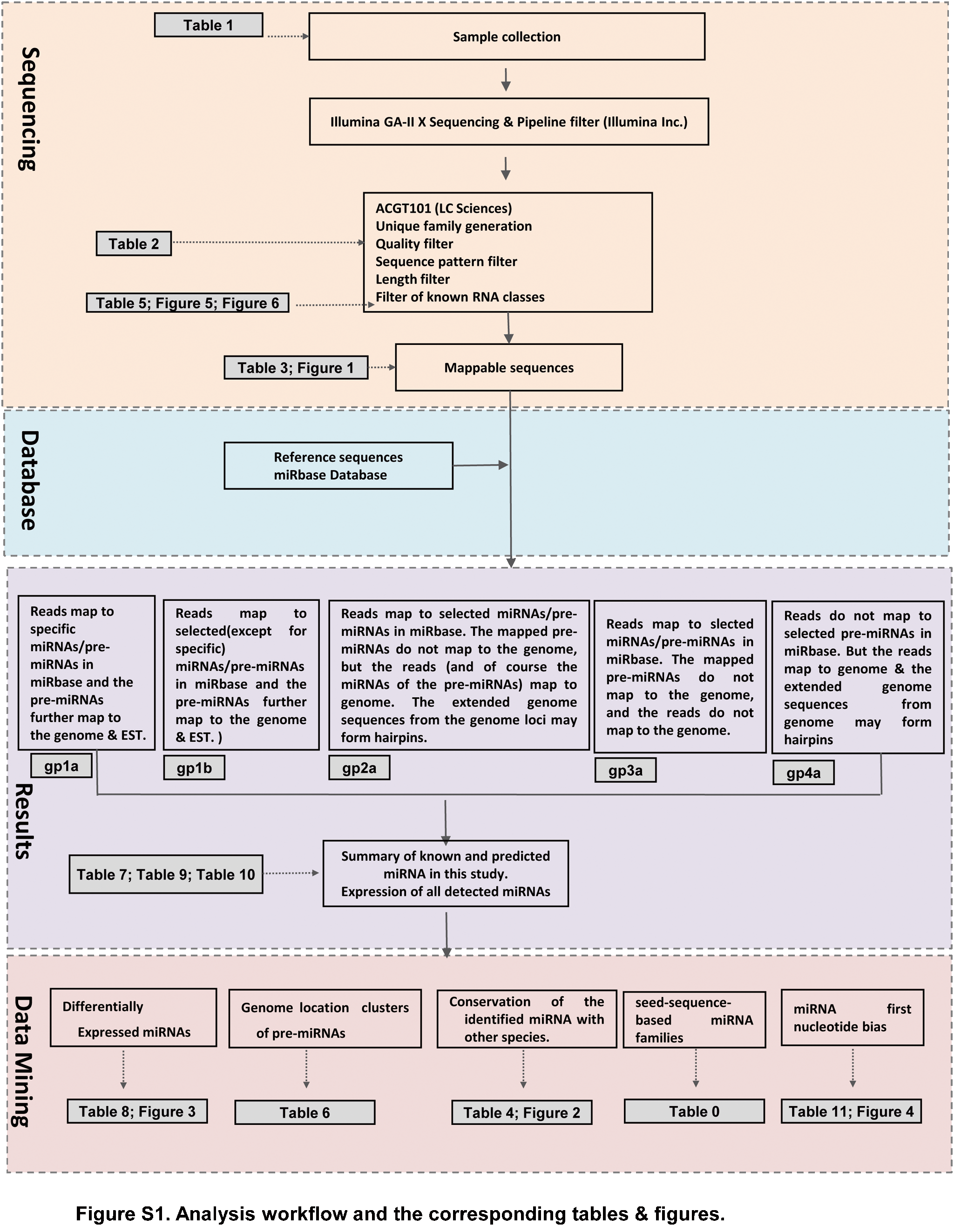

Supplement: Figure S1 — A flow-chart of study procedures. (TIF) [file pone.0107986.s001.tif]
