# Supplementary material for: A Serum MicroRNA Panel as Potential Biomarkers for Hepatocellular Carcinoma Related with Hepatitis B Virus
Source: PLoS One. 2014 Sep 19;9(9):e107986. doi: 10.1371/journal.pone.0107986 (PMC4169601; doi:10.1371/journal.pone.0107986)
Supplement: Table S1 — Overview of reads from raw data to cleaned sequences. (DOCX) [file pone.0107986.s002.docx]

| Table S1 Overview of reads from raw data to cleaned sequences | | | | | | | | | | | | | |
| --- | --- | --- | --- | --- | --- | --- | --- | --- | --- | --- | --- | --- | --- |
| lib | type | Healthy | | | | Cirrhosis | | | | HCC | | | |
|  |  | Total | % of Total | uniq | % of uniq | Total | % of Total | uniq | % of uniq | Total | % of Total | uniq | % of uniq |
| Raw reads | NA | 9364754 | 100 | 895564 | 100 | 10491694 | 100 | 1433485 | 100 | 7896608 | 100 | 906910 | 100 |
| 3ADT&length filter | Sequence type | 956376 | 10.21 | 245676 | 27.43 | 1058204 | 10.09 | 416413 | 26.21 | 600175 | 7.6 | 298902 | 29.93 |
| Junk reads | Sequence type | 6853 | 0.07 | 2464 | 0.28 | 7254 | 0.07 | 3130 | 0.22 | 4244 | 0.05 | 2097 | 0.23 |
| Rfam | RNA class | 697537 | 7.45 | 55485 | 6.20 | 728242 | 6.94 | 86354 | 6.02 | 451536 | 5.72 | 67072 | 7.4 |
| mRNA | RNA class | 384765 | 4.11 | 60756 | 6.78 | 399759 | 3.81 | 84910 | 5.92 | 255154 | 3.23 | 56120 | 6.19 |
| Repeats | RNA class | 85473 | 0.91 | 8956 | 1.00 | 85703 | 0.82 | 12351 | 0.86 | 81965 | 1.04 | 7919 | 0.87 |
| rRNA | RNA class | 317546 | 3.39 | 31543 | 0.43 | 331330 | 3.16 | 44678 | 0.43 | 203358 | 2.58 | 36844 | 0.47 |
| tRNA | RNA class | 157433 | 1.68 | 11465 | 1.28 | 180585 | 1.72 | 17355 | 0.17 | 140149 | 1.77 | 13985 | 0.18 |
| snoRNA | RNA class | 20754 | 0.22 | 4532 | 0.51 | 22776 | 0.22 | 5645 | 0.05 | 18667 | 0.24 | 3936 | 0.05 |
| snRNA | RNA class | 26453 | 0.28 | 3154 | 0.35 | 28328 | 0.27 | 4234 | 0.04 | 22867 | 0.29 | 2709 | 0.03 |
| other Rfam RNA | RNA class | 85674 | 0.91 | 12643 | 1.41 | 165223 | 1.57 | 14442 | 0.14 | 66495 | 0.84 | 9598 | 0.12 |
| Clean reads | Sequence type | 6625890 | 77.75 | 459890 | 52.98 | 8418846 | 80.24 | 859216 | 59.94 | 6677304 | 84.56 | 494523 | 54.53 |
