# Supplementary material for: A Serum MicroRNA Panel as Potential Biomarkers for Hepatocellular Carcinoma Related with Hepatitis B Virus
Source: PLoS One. 2014 Sep 19;9(9):e107986. doi: 10.1371/journal.pone.0107986 (PMC4169601; doi:10.1371/journal.pone.0107986)
Supplement: Table S2 — AUC of ROC curves between HCC and healthy controls in the training set. (DOCX) [file pone.0107986.s003.docx]

| Table S2 AUC of ROC curves between HCC and healthy controls in training set | | | | | | |
| --- | --- | --- | --- | --- | --- | --- |
| Variable | AUC | 95% CI | Sencitivity | Specificity | z statistic | *p* |
| hsa_miR_206 | 0.615 | 0.548 to 0.683 | 48.1 | 78.8 | 3.344 | <0.0001 |
| hsa-miR-141-3p | 0.758 | 0.695 to 0.822 | 68.1 | 83.3 | 7.938 | <0.0001 |
| hsa_miR_433_5p | 0.736 | 0.668 to 0.804 | 79.3 | 64.4 | 6.769 | <0.0001 |
| hsa-miR-1228-5p | 0.552 | 0.444 to 0.6 | 79.3 | 27.8 | 0.555 | 0.5792 |
| hsa-miR-199a-5p | 0.638 | 0.565 to 0.712 | 59.3 | 66.7 | 3.694 | 0.0002 |
| hsa-miR-122-5p | 0.697 | 0.628 to 0.766 | 48.9 | 82.2 | 5.575 | <0.0001 |
| hsa-miR-192-5p | 0.695 | 0.624 to 0.766 | 71.9 | 75.6 | 5.402 | <0.0001 |
| hsa-miR-26a-5p | 0.762 | 0.700 to 0.824 | 68.9 | 74.4 | 8.321 | <0.0001 |
|  |  |  |  |  |  |  |
