# Supplementary material for: A Serum MicroRNA Panel as Potential Biomarkers for Hepatocellular Carcinoma Related with Hepatitis B Virus
Source: PLoS One. 2014 Sep 19;9(9):e107986. doi: 10.1371/journal.pone.0107986 (PMC4169601; doi:10.1371/journal.pone.0107986)
Supplement: Table S3 — AUC of ROC curves between HCC and cirrhosis in the training set. (DOCX) [file pone.0107986.s004.docx]

| Table S3 AUC of ROC curves between HCC and cirrhosis in training set set | | | | | | |
| --- | --- | --- | --- | --- | --- | --- |
| Variable | AUC | 95% CI | Sencitivity | Specificity | z statistic | *p* |
| hsa_miR_206 | 0.693 | 0.621 to 0.766 | 77.8 | 68.9 | 5.224 | 0.008 |
| hsa-miR-141-3p | 0.663 | 0.596 to 0.730 | 60.7 | 72.7 | 4.776 | <0.0001 |
| hsa_miR_433_5p | 0.644 | 0.578 to 0.710 | 56.4 | 67.4 | 4.294 | <0.0001 |
| hsa-miR-1228-5p | 0.542 | 0.470 to 0.611 | 66.7 | 47 | 1.178 | 0.239 |
| hsa-miR-199a-5p | 0.589 | 0.521 to 0.657 | 59.3 | 57.6 | 2.556 | 0.0106 |
| hsa-miR-122-5p | 0.751 | 0.693 to 0.809 | 48.9 | 90.2 | 8.521 | <0.0001 |
| hsa-miR-192-5p | 0.687 | 0.623 to 0.751 | 54.8 | 83.3 | 5.742 | <0.0001 |
| hsa-miR-26a-5p | 0.744 | 0.680 to 0.807 | 60.7 | 90.9 | 7.627 | <0.0001 |
|  |  |  |  |  |  |  |
