# Supplementary material for: A Serum MicroRNA Panel as Potential Biomarkers for Hepatocellular Carcinoma Related with Hepatitis B Virus
Source: PLoS One. 2014 Sep 19;9(9):e107986. doi: 10.1371/journal.pone.0107986 (PMC4169601; doi:10.1371/journal.pone.0107986)
Supplement: Table S4 — AUC of ROC curves between HCC and control in the training set. (DOCX) [file pone.0107986.s005.docx]

| Table S4 AUC of ROC curves between HCC and control in training set set | | | | | | |
| --- | --- | --- | --- | --- | --- | --- |
| Variable | AUC | 95% CI | Sencitivity | Specificity | z statistic | *p* |
| hsa_miR_206 | 0.665 | 0.607 to 0.724 | 85.2 | 52.3 | 5.527 | <0.0001 |
| hsa-miR-141-3p | 0.68 | 0.617 to 0.742 | 60.7 | 77 | 5.633 | <0.0001 |
| hsa_miR_433_5p | 0.607 | 0.548 to 0.666 | 83 | 39.2 | 3.56 | 0.0004 |
| hsa-miR-1228-5p | 0.534 | 0.472 to 0.596 | 66.7 | 43.7 | 1.071 | 0.2843 |
| hsa-miR-199a-5p | 0.609 | 0.549 to 0.669 | 59.3 | 61.3 | 3.563 | 0.0004 |
| hsa-miR-122-5p | 0.729 | 0.675 to 0.784 | 48.9 | 86.9 | 8.227 | <0.0001 |
| hsa-miR-192-5p | 0.69 | 0.630 to 0.750 | 54.8 | 84.7 | 6.221 | <0.0001 |
| hsa-miR-26a-5p | 0.677 | 0.614 to 0.739 | 60.7 | 75.2 | 5.505 | <0.0001 |
|  |  |  |  |  |  |  |
