# Supplementary material for: A Serum MicroRNA Panel as Potential Biomarkers for Hepatocellular Carcinoma Related with Hepatitis B Virus
Source: PLoS One. 2014 Sep 19;9(9):e107986. doi: 10.1371/journal.pone.0107986 (PMC4169601; doi:10.1371/journal.pone.0107986)
Supplement: Table S5 — Logistic regression of miRNAs between HCC patients and control subjects in the training set. (DOCX) [file pone.0107986.s006.docx]

| Table S5 Logistic regression of miRNAs between HCC and control in training dataset | | | | | |
| --- | --- | --- | --- | --- | --- |
| Variable | Coefficient | Std. Error | Odds ratio | 95% CI | P |
| hsa_miR_122_5p | 0.52147 | 0.10012 | 1.6845 | 1.3844 to 2.0497 | <0.0001 |
| hsa_miR_1228_5p | -0.22949 | 0.09033 | 0.7949 | 0.6660 to 0.9489 | 0.011 |
| hsa_miR_141_3p | -0.27621 | 0.09116 | 0.7587 | 0.6345 to 0.9071 | 0.002 |
| hsa_miR_192_5p | 0.34063 | 0.09948 | 1.4058 | 1.1568 to 1.7085 | 6E-04 |
| hsa_miR_199a_5p | 0.33325 | 0.09563 | 1.3955 | 1.1570 to 1.6832 | 5E-04 |
| hsa_miR_206 | -0.30556 | 0.08214 | 0.7367 | 0.6272 to 0.8654 | 2E-04 |
| hsa_miR_26a_5p | 0.40777 | 0.08695 | 1.5035 | 1.2679 to 1.7828 | <0.0001 |
| hsa_miR_433_3p | -0.38006 | 0.11592 | 0.6838 | 0.5448 to 0.8582 | 0.001 |
| Constant | -11.8472 |  |  |  |  |
| Enter variable if P< 0.05 ,remove variable if P> 0.1;Overall model fit:Null model -2 Log Likelihood =473.49 ;Full model -2 Log Likelihood= 289.255 ;*x*^2^ =184.246 ,*P* < 0.0001.  logitP=-11.8472+0.52147miR122-0.22949miR1228-0.27621miR141+0.34063miR192+0.33325mi199a-0.30556miR206+0.40777miR26a-0.38006miR433 | | | | | |
|  |  |  |  |  |  |
|  |  |  |  |  |  |
|  |  |  |  |  |  |
|  |  |  |  |  |  |
|  |  |  |  |  |  |
|  |  |  |  |  |  |
